# Prolapse of a large pedunculated uterine myoma

**DOI:** 10.1002/ccr3.5393

**Published:** 2022-02-15

**Authors:** Ioannis K. Papapanagiotou, Sofoklis Stavros, Dimitrios‐Efthymios Vlachos, Kyriaki Migklis, Konstantina Papadatou, Dimitrios Papageorgiou, Stamo Manouvelou, Athanasios Zikopoulos, Ekaterini Domali, Peter Drakakis, Alexandros Rodolakis, Nikolaos Thomakos

**Affiliations:** ^1^ 1st Department of Obstetrics and Gynecology General Hospital “Alexandra” Athens Greece; ^2^ Department of Radiology General Hospital “Alexandra” Athens Greece; ^3^ Royal Cornwall Hospital Truro UK

**Keywords:** hysterectomy, leiomyoma, pedunculated, uterine bleeding

## Abstract

We report a rare case of a large prolapsed pedunculated uterine myoma measuring 15 cm in its greater diameter. In order to make a surgical procedure safe and feasible, appropriate clinical predictors should be taken into account and pre‐ and intraoperative preparations be available to the surgeon's armamentarium.

## Graphical abstract

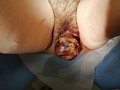

## 1 QUESTION

What is this condition and how can it be treated?

## 2 ANSWER

**FIGURE 1:**
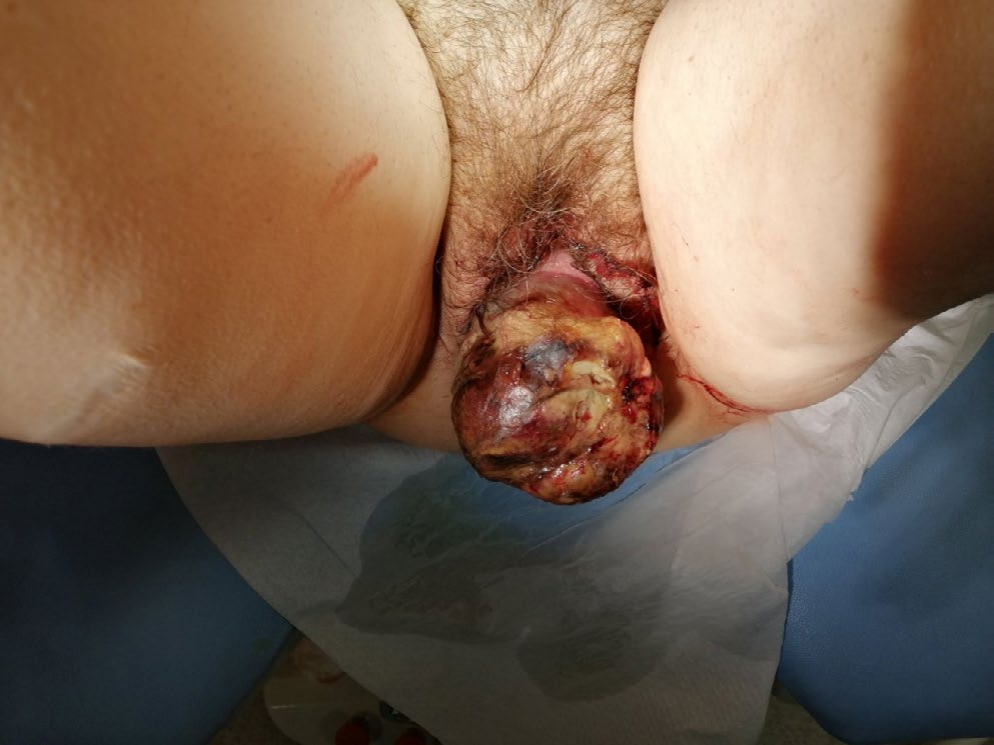
Digital rectal examination exposed a very large sphincteric defect, while both the resting tone and the squeeze contraction were completely absent

**FIGURE 2:**
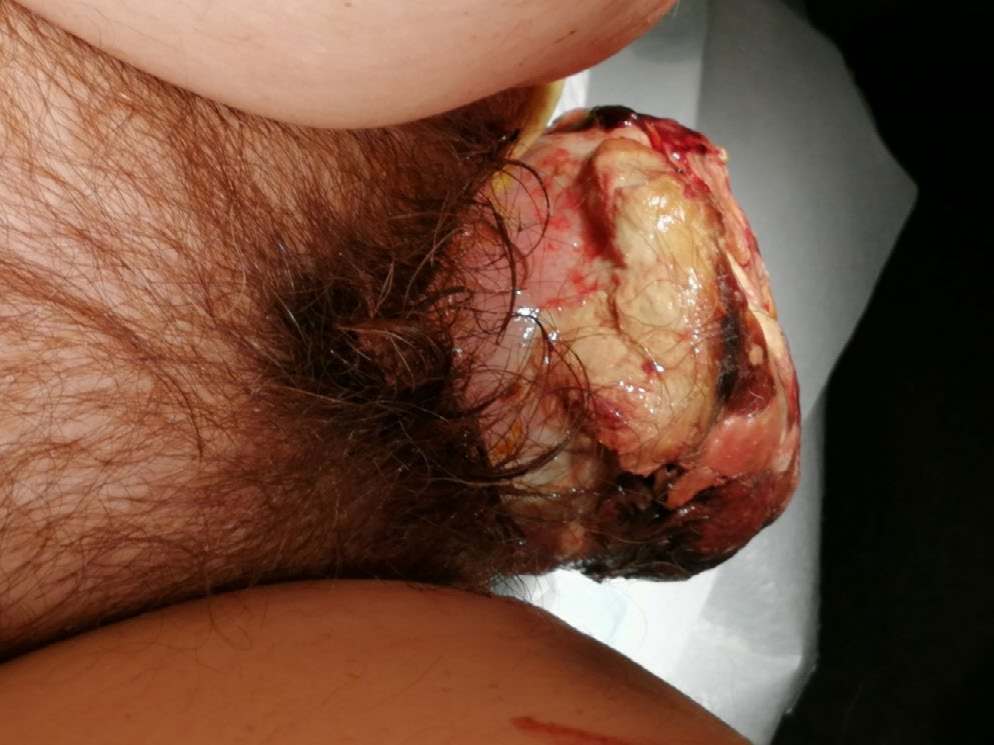
Anal inspection revealed the absence of the perineal body, the corrugator cutis ani, and an off‐site aperture of the anal canal in the posterior proximal vaginal surface

**FIGURE 3:**
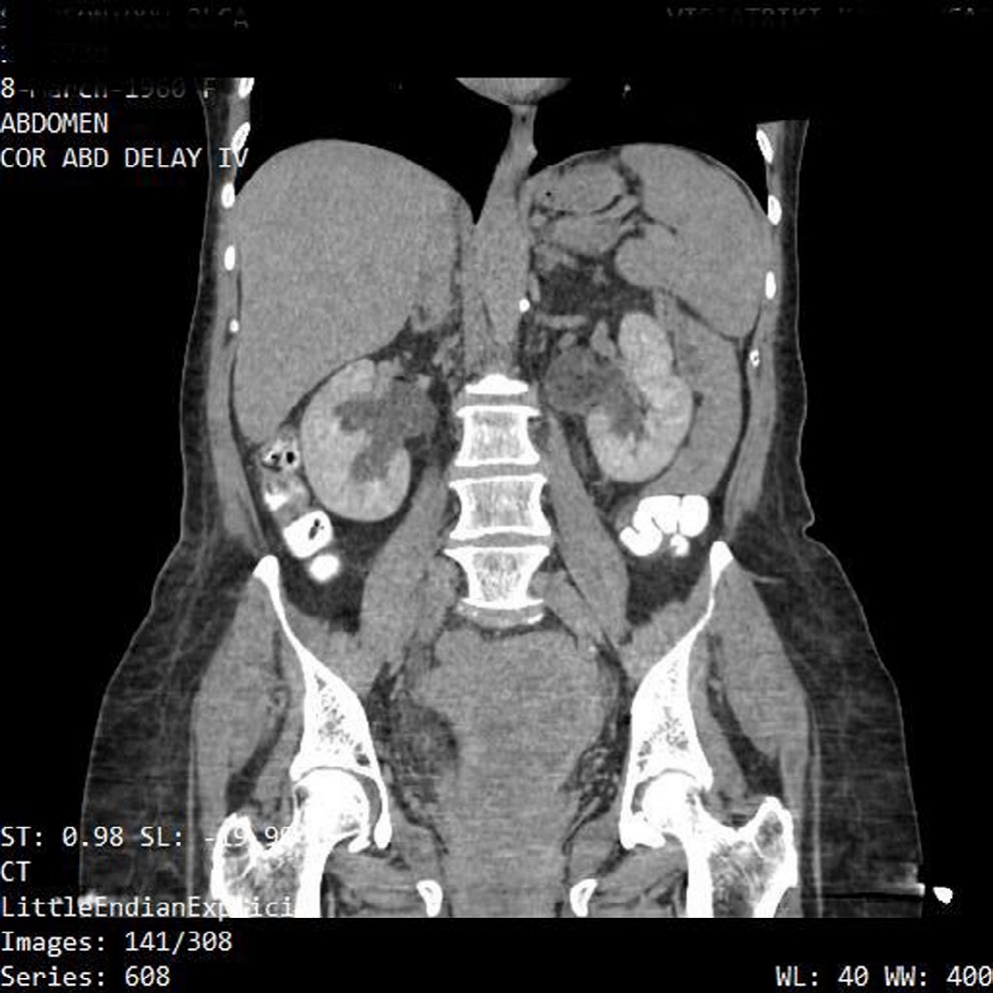
Abdominal CT image in delayed post contrast venous phase, depicting a mildly hypo‐dense lesion with indistinct boarders, and extending from the cervix to the endometrium and the vagina. Mild bilateral hydronephrosis of the kidneys is also noted

The clinical image demonstrates a large pedunculated uterine myoma in a 60‐year‐old postmenopausal woman. The patient complained for a vulvar protruding mass, vaginal bleeding, and abdominal pain. Clinical examination revealed a neglected mass originating above the level of the internal cervical os (Figures 1 and 2). Blood laboratory values were normal. Computed tomography examination revealed a uterine mass measuring 15 cm × 10 cm × 7 cm with bilateral hydronephrosis (Figure 3). The patient underwent abdominal hysterectomy and bilateral salpigoophorectomy. Biopsies retrieved from the mass were negative for malignancy. Pathological report of the surgical specimen indicated a leiomyoma with ulcerative, necrotic, vitreous, and cystic degenerative areas.

Uterine leiomyoma are the most common pelvic tumors in women. Reported prevalence of prolapsed pedunculated submucosal leiomyoma is 2.5%.^1^ Most myomas are small but variable in size, between 1 cm and 6 cm; however, some case reports in the literature have reported prolapse of larger myomas, measuring more than 10 cm in diameter.^2^

Surgery has been the mainstay of prolapsed pedunculated leiomyomas, with both vaginal removal and hysterectomy being safe.^1^ Lower parity, absence of coexisting leiomyoma, low volume of leiomyoma, and more severe anemia were associated with preference of vaginal removal.^1^ On the contrary, hysterectomy is preferred in postmenopausal women with multiple leiomyomas or in cases of larger volume.

## CONFLICT OF INTEREST

None.

## AUTHOR CONTRIBUTIONS

I.K.P. has made substantial contribution to conception, analyzing, and drafting the manuscript. S.S. has made substantial contribution to acquisition of data. D‐E.V. has contributed in analyzing data and revising the manuscript. K.M. has contributed in acquisition of the data. K.P. and D.P. has contributed in acquisition of the data. S.M. has processed the radiological images. E.D. and A.Z. have contributed in analyzing data. P.D. has agreed to be accountable for all aspects of the work. A.R. has given final approval of the version to be published. N.T. has revised the manuscript.

## ETHICAL APPROVAL

Patient consent has been collected. The ethics committee of the hospital has approved this clinical image.

## CONSENT

Written informed consent was obtained from the patient to publish this report in accordance with the journal's patient consent policy.

## Data Availability

Data sharing is not applicable—no new data generated—the article describes entirely an obstetrical complication.
